# Structural Insights into Omega-Class Glutathione Transferases: A Snapshot of Enzyme Reduction and Identification of a Non-Catalytic Ligandin Site

**DOI:** 10.1371/journal.pone.0060324

**Published:** 2013-04-09

**Authors:** Joseph Brock, Philip G. Board, Aaron J. Oakley

**Affiliations:** 1 Research School of Chemistry, Australian National University, Canberra, Australian Capital Territory, Australia; 2 John Curtin School of Medical Research, Australian National University, Canberra, Australian Capital Territory, Australia; 3 School of Chemistry, University of Wollongong, New South Wales, Australia; University of Queensland, Australia

## Abstract

Glutathione transferases (GSTs) are dimeric enzymes containing one active-site per monomer. The omega-class GSTs (hGSTO1-1 and hGSTO2-2 in humans) are homodimeric and carry out a range of reactions including the glutathione-dependant reduction of a range of compounds and the reduction of S-(phenacyl)glutathiones to acetophenones. Both types of reaction result in the formation of a mixed-disulfide of the enzyme with glutathione through the catalytic cysteine (C32). Recycling of the enzyme utilizes a second glutathione molecule and results in oxidized glutathione (GSSG) release. The crystal structure of an active-site mutant (C32A) of the hGSTO1-1 isozyme in complex with GSSG provides a snapshot of the enzyme in the process of regeneration. GSSG occupies both the G (GSH-binding) and H (hydrophobic-binding) sites and causes re-arrangement of some H-site residues. In the same structure we demonstrate the existence of a novel “ligandin” binding site deep within in the dimer interface of this enzyme, containing S-(4-nitrophenacyl)glutathione, an isozyme-specific substrate for hGSTO1-1. The ligandin site, conserved in Omega class GSTs from a range of species, is hydrophobic in nature and may represent the binding location for tocopherol esters that are uncompetitive hGSTO1-1 inhibitors.

## Introduction

The inducible phase II enzymes known as glutathione transferases (GSTs; E.C. 2.5.1.18) conjugate endogenous and xenobiotic toxins with electrophilic centers to glutathione (γ-glu-cys-gly, GSH). Several classes function as glutathione peroxidases or as reductases [1]. Among the human isozymes are the cytoplasmic alpha, zeta, theta, mu, pi, sigma and omega classes. The most recently described family in humans is omega: two isozymes have been identified (designated hGSTO1-1 and hGSTO2-2) [2], [3]. The Omega class GSTs are associated with biological processes including the modulation of ryanodine receptors [4] and the activation of IL-1β [5]. Polymorphisms in the Omega class GSTs have been associated with the age at onset of Alzheimer’s and Parkinson’s diseases [6], familial amyotrophic lateral sclerosis [7], and the development of acute childhood lymphoblastic leukemia [8].

Like all cystosolic GSTs, the omega-class isozymes have an N-terminal thioredoxin-like domain and a unique helical C-terminal domain [9], [10]. The active sites of most GSTs contain a serine or tyrosine hydroxyl group that promotes the ionization of the GSH sulfhydryl group. The omega-class isozymes instead have a cysteine residue (C32 in hGSTO1 and O2) in the active site that is oxidized through the formation of an enzyme-GSH mixed disulfide with the concomitant reduction of a co-substrate. Thus the omega class isozymes function as thiol transferases/reductases. Reactions catalysed include dehydroascorbate reduction and monomethylarsenate reduction [2], [10]–[12].

**Figure 1:**
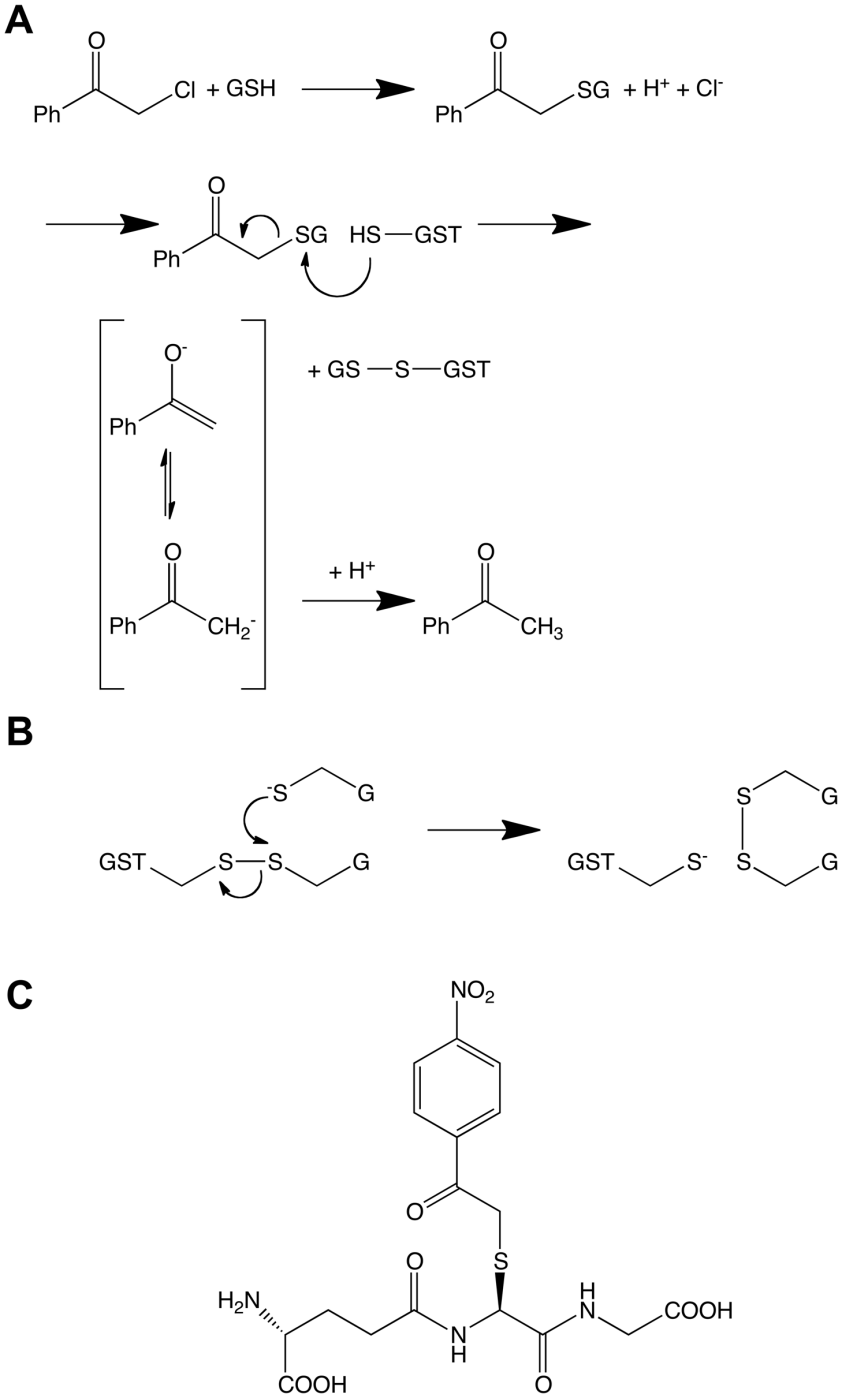
Chemical reactions and species. (A) Proposed reaction mechanisms for the (non enzymatic) formation of S-(phenacyl) glutathiones and their (hGSTO1-1-catalyzed) reduction to acetophenones, and (B) the reduction of oxidised hGSTO1-1 by a second molecule of GSH. (C) chemical structure of 4NPG.

Recently the role of omega-class GSTs in the disposition of α-haloketones has been investigated. The α-haloketones are a class of biologically active compounds that can enter the human body *via* several pathways. Some α-haloketones have been identified as metabolites of insecticides [13]. 2-Chloroacetophenone is an α-haloketone used as a temporary incapacitating agent in tear-gas. The non-enzymatic attack by GSH upon 2-chloroacetophenone gives rise to S-(phenacyl)glutathione, which in turn is decomposed reductively by hGSTO1-1 [14]. In contrast to other known activities of the omega class GSTs, this reaction is unique to hGSTO1-1 as hGSTO2-2 fails to show appreciable activity to this class of substrate. This mechanism is thought to operate via nucleophilic attack of the active site cysteine upon the cysteinyl sulfur of the S-(phenacyl)glutathione, releasing acetophenone and forming a mixed disulfide with the GSH moiety (Figure 1A). Physiologically, the enzyme is regenerated by the nucleophilic attack of a second GSH molecule upon the mixed disulfide, reducing the active-site cysteine and producing oxidized glutathione (GSSG) (Figure 1B). β-Mercaptoethanol can substitute for the second GSH for the regeneration of hGSTO1-1, increasing the catalytic rate constant (*k*_cat_) by a factor of five [14]. A new compound, S-(4-nitrophenacyl)glutathione (4NPG) (Figure 1C), has recently been synthesised that has a turnover rate that is approximately 15 times higher, and displays a catalytic efficiency more than 200 times higher than previously observed with S-(phenacyl)glutathione [15]. In addition, it allows hGSTO1-1 activity to be measured spectrophotometrically by a characteristic absorbance change at 305 nm.

In addition to activities involving GSH and its conjugates, several classes of GST have been shown to exhibit “ligandin” activity, i.e., non-catalytic ligand binding. In the case of a squid sigma- and a blood fluke mu-class GST, this has been demonstrated to occur in the dimer interface [16], [17]. In the human pi-class GST, the ligandin site occupies part of the H-site [18]. To date, no ligandin binding site has been structurally characterized in an omega-class GST.

In this report, we have describe a crystal structure in which GSSG is observed in the active site of an inactive hGSTO1-1 mutant (C32A), giving us a snapshot of enzyme regeneration occurring. The same structure reveals the binding of 4NPG in the dimer interface, revealing a non-catalytic ligandin binding site.

## Methods

Protein was purified as described previously [15]. Briefly, the hGSTO1-1 C32A mutant was expressed in *Escherichia coli* BL21 (DE3) cells as an N-terminal poly-His-tagged ubiquitin fusion protein from the pHUE plasmid [19]. An initial purification step on Ni-NTA agarose was followed by cleavage by a modified mouse deubiquitylating enzyme [20] to yield enzyme with no additional N-terminal residues. A second pass over Ni-NTA agarose gave pure protein. In these experiments 5 mM DTT was substituted with 1 mM TCEP for the reducing agent in the final dialysis buffer in order to prevent auto cleavage of the substrate in subsequent crystal soaking experiments via formation of a GSH-DTT mixed disulfide, in a manner analogous to the reaction with β-mercaptoethanol described above. Datasets were collected from two crystals grown under similar conditions. Both were grown via the hanging-drop vapour diffusion method at 4°C. The reservoir consisted of 2.2 M ammonium sulfate and 100 mM sodium acetate pH 4.25 and 4.75 respectively. Crystallization drops contained 1 µl hGSTO1-1 C32A at 32 mg/ml combined with 1 µl of reservoir solution. The crystals were then transferred to pre-equilibrated soaking drops containing 2 µl of reservoir solution and 2 µl of 10 mM 4NPG pH 7.0. Prior to this transfer, one of the crystals was also soaked in a drop containing 2 µl of reservoir together with 0.5 µl of GSH pH 7.5. Crystals were subsequently cryoprotected via stepwise transfer to artificial mother liquor containing 2.75 M lithium sulfate, 100 mM sodium acetate pH 4.75 and glycerol at up to 15% (v/v).

Data was subsequently collected remotely at the SSRL using an X-ray wavelength of 1.034375 Å (12 keV). X-ray data was processed using software within the CCP4 suite [21]: the diffraction images were processed and integrated using the programs MOSFLM and SCALA. After phasing each dataset separately using previously published complex with GSH (PDB code: 1EEM) it was found that in spite of the slight differences in soaking conditions, no significant differences could be observed in the 2m*F*_O_-D*F*_C_ or m*F*_O_-D*F*_C_ electron density maps. POINTLESS was therefore used to combine the two datasets and ascertain their Laue symmetry before scaling with SCALA. The starting model for refinement was again the previously published structure, (PDB code: 1EEM) [2]. Molecular modelling of ligand into m*F*_O_-D*F*_C_ density was performed with COOT [22]. Ligand restraint generation and structure refinement was performed with Phenix [23]. The coordinates and X-ray structure factor amplitudes have been deposited with the PDB (ID: 4IS0).

## Results

**Figure 2:**
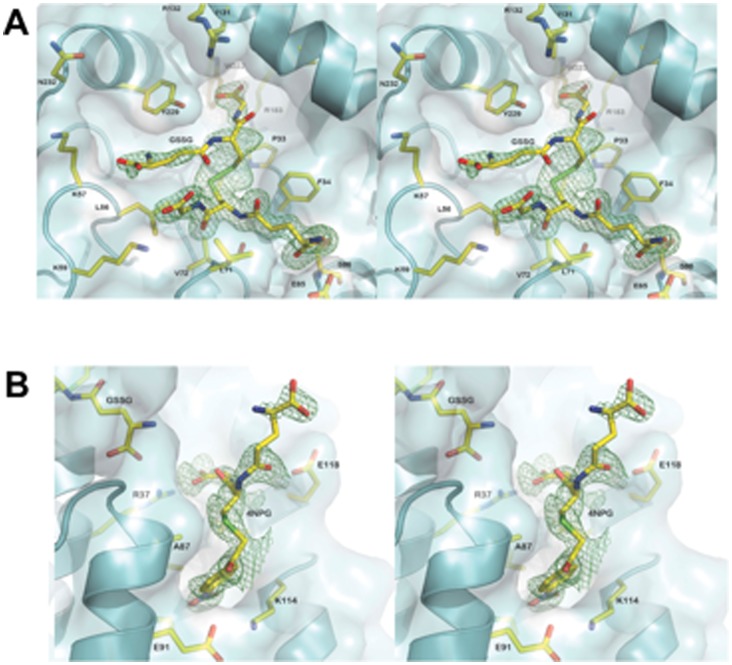
Electron density omit-maps of ligands. Binding sites in hGSTO1-1 for (A) GSSG and (B) the 4NPG are shown. The chemical entities and surrounding residues are in stick representation. Electron density maps (m*F*_O_-D*F*_C_) calculated in Phenix are shown in green, contoured at 3 σ. The enzyme is shown in cartoon form.

**Figure 3:**
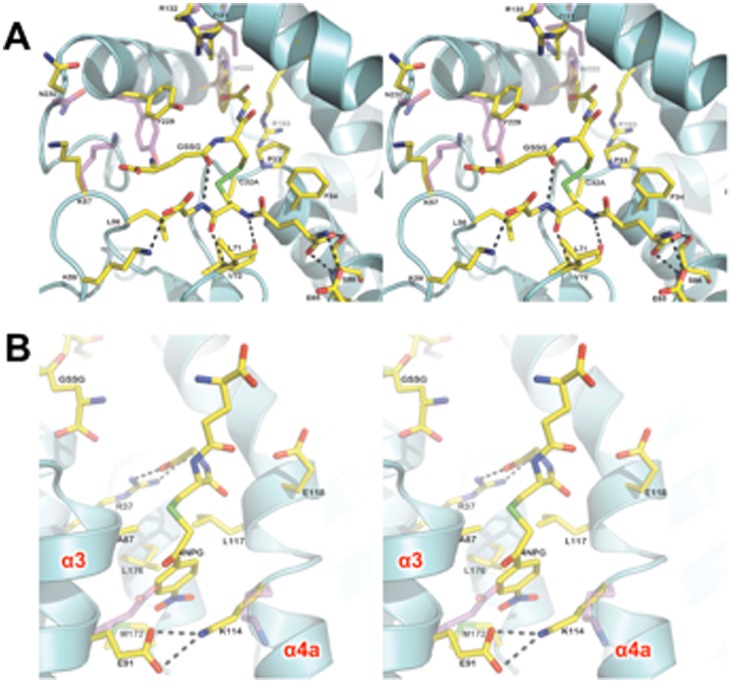
hGSTO1-1 ligand structure. The fold of hGSTO1-1 in complex with GSSG/4NPG is shown as a cartoon representation (cyan) with ligands and amino acid residues shown in stick representation, coloured according to atom type. Side chains of significantly different conformation within the GSH complex (PDB id: 1EEM) are overlayed (magenta carbon atoms). Polar interactions are shown with black dashed lines. (A) The active site of hGSTO1-1, showing the complex of GSSG and associated conformational change in the “H-site”. (B) The ligandin-binding site of hGSTO1-1 as viewed from the point of view of the opposing monomer, which has been removed for clarity.

The final structure contains one protomer (residues 4 to 241), two sulfate molecules, one each of 4NPG, GSSG and DTT molecules. A total of 140 water molecules were built into the model. The asymmetric unit contains a single monomer: the physiologically relevant dimer is produced by two-fold crystallographic symmetry. The crystals of hGSTO1-1 C32A mutant are similar to that reported for the wild-type enzyme [2] (Table 1), superimposing with a RMSD of 0.30 Å over 237 Cα atoms. Our attempt to determine the structure of hGSTO1-1 in complex with 4NPG has revealed GSSG bound in the active site and 4NPG bound at the dimer interface (Figure 2). The likely source of GSSG is the non-enzymatic reaction of 4NPG with residual GSH in the crystallization mixture. The GSSG dimer binds with one half of the molecule in the G-site, with interactions the same as those observed for reduced glutathione binding. The other half of the GSSG dimer extends upwards into the H-site and is less well ordered (Figure 2A). Indeed, interactions with this half of the ligand are observed to be exclusively hydrophobic in character, with only an internal hydrogen bond observed between the γ-glutamyl carbonyl and the glycinyl-amine of the G-site bound half of the molecule. The lack of well-defined interactions with the protein undoubtedly contributes to the relatively poor electron density and high B-factors associated with the portion of the molecule in the H-site. The binding of GSSG is associated with the structural rearrangement of several amino acid side chains relative to the previously published complex with glutathione [2]. H-site residue Y229 has shifted to accommodate the γ-glutamyl residue and the indole group of W222 has rotated 180° (Figure 3A). In addition, the nearby side chains of K57, I131 and R132 are relatively poorly ordered.

**Table 1:** Crystallographic statistics.

**Diffraction data**	
Space group	P3_1_21
Unit cell Dimensions (Å,°)	a = 57.6, b = 57.6, c = 140.2, α = 90.0, β = 90.0, γ = 120.0
Resolution limits (Å)	40.64−1.72 (1.81−1.72)†
Unique reflections	29,422 (4,197)
Completeness (%)	99.9 (99.1)
Multiplicity	16.2 (7.2)
R-merge (%)	8.1 (60.9)
I/σI	19.2 (2.3)
**Refinement data**	
R-factor (%)	15.19
R-free (%)	20.07
RMSD from ideal geometry:	
Bonds	0.011 Å
Angles	1.424°
Chiral volumes	0.071 Å^3^
Planar groups	0.007 Å

† Values in parentheses refer to the highest resolution bin.

**Figure 4:**
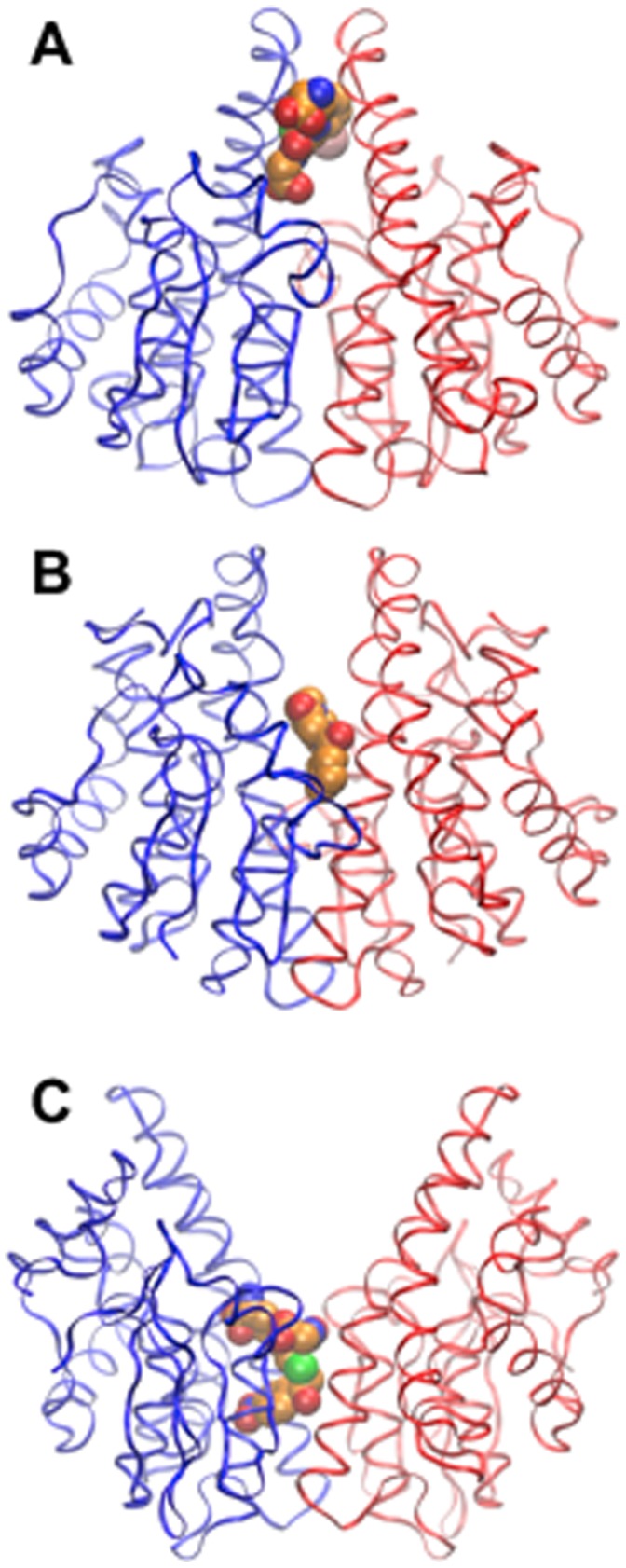
Structures of GSTs with ligands bound in the dimer interface. Monomers are shown as ribbons, ligands as van der Waals surfaces. (A) Squid sigma-class GST with S-(3-iodobenzyl)glutathione [17] (PDB ID: 2GSQ). (B) praziquantel to a mu-class GST of the parasitic worm *Schistosoma japonica* (PDB ID: 1GTB) [16] (C) 4NPG bound to hGSTO1-1 (this work).

**Figure 5:**
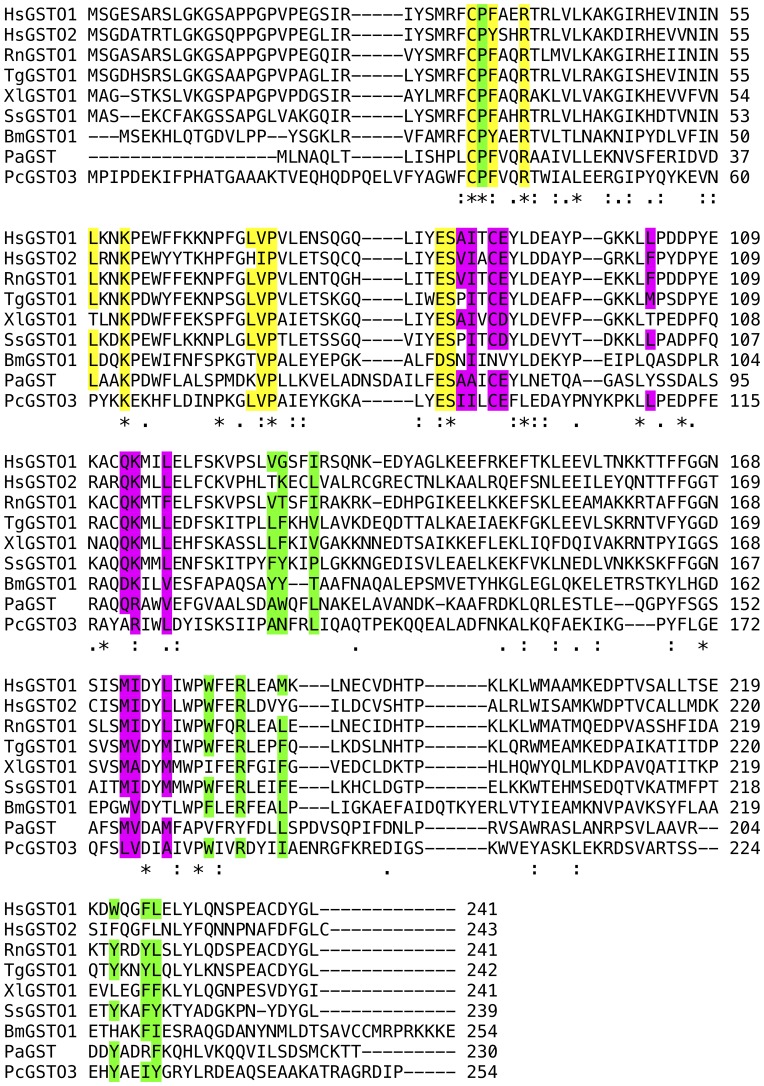
Sequence alignment of representative hGSTO1 homologues. The species and genbank identifiers of the sequences are Hs (*Homo sapiens*, O1 gi: 4758484; O2 gi: 34922124) Rn (*Rattus norvegicus*, gi: 56090550), Tg (*Taeniopygia guttata*, gi: 224052779), Xl (*Xenopus laevis*, gi: 147907264), Ss (*Salmo salar*, gi: 213511516), Bm (*Bombyx mori*, gi: 87248151), Pa (*Pectobacterium atrosepticum*, gi: 50120521), Pc (*Phanerochaete chrysosporium*, gi: 193161505). Conserved or conservatively substituted residues are highlighted in yellow (G-site), green (H-site residues contacting the second GSH moiety), and magenta (L-site residues in the dimer interface).

4NPG binds in the dimer interface, deep within the cleft formed between monomers. Because it sits on a crystallographic two-fold axis, the electron density corresponds to two overlapping 4NPG molecules at half-occupancy (Figure 2B). The glutathionyl component of the molecule is relatively disordered. The bulk of interactions of the protein are with the nitrophenacyl moiety (Figure 3B). An exception is the glycinyl moiety of the compound, observed adjacent to the γ-glutamyl tail of GSSG, engaging in a salt bridge interaction with the side chain of R37. The nitrophenacyl functional group is observed pointing downwards into the dimeric cleft. The binding site is too far from the active site to be of catalytic relevance (the distance between the mutated active-site C32A residue and 4NPG sulfur atom is about 17 Å). The 4NPG-binding site is largely hydrophobic, lined by residues from helix α3 (A87, I88, C90, E91), the following loop (L103), helix α4 (Q113, K114, L117) and helix α6 (M172, I173, L176). The bottom of the pocket is formed by E91 and K114, which form a salt bridge interaction. Relative to the structure of wild-type hGSTO1-1 without ligand bound in the dimer interface, side-chain movements are seen in K114 and E91, which move closer so as to bind 4NPG with their aliphatic moieties and form the salt bridge interaction. The binding mode of 4NPG in the dimer interface may be representative of a ligandin-binding site similar to that observed in other classes of GST. The binding of the anti-Schistosomiasis drug Praziquantel to a mu-class GST from the parasitic worm *Schistosoma japonica* [16], and the complex formation of the GSH-conjugate, S-(3-iodobenzyl)glutathione with a sigma-class GST of squid [17] are both reminiscent of the dimer interface mode of binding observed for 4NPG (Figure 4). The residues lining the binding site are well conserved across GSTO homologues from a range of species (Figure 5).

## Discussion

Some members of the GST family of enzymes were originally identified as “ligandins”, due to their apparent capacity to bind a wide variety of large (>400 Da) lipophilic compounds such as bile acids, fatty acids and certain drugs. This function was thought to play a role in storage and transport of these compounds in the aqueous phase of the cell [24]. The position of several of these ligandin or “L-site” binding pockets have been identified crystallographically in a broad spectrum of GSTs. While their positions within the phi-class GST of *Arabidopsis thaliana* [25] and the human pi-class GST [18] were observed to overlap with the H-site, this is not always the case. The binding of the anti-Schistosomiasis drug praziquantel to a mu-class GST of the parasitic worm *Schistosoma japonicum* [16], S-(3-iodobenzyl)glutathione to a squid sigma-class GST [17], and now, 4NPG to hGSTO1-1 occur in the dimer interface and straddles the two-fold axis. As can be observed in Figure 4, the location of the ligand along the two-fold axis appears to be related to the width of the interface: hGSTO1-1 has the widest interface and the deepest ligandin-site of these GSTs. This ligandin site in hGSTO1-1 may be the binding site for non-competitive inhibitors. (+)-α-Tocopherol succinate has been reported to be a non-competitive inhibitor of the monomethylarsonate (V) reductase activity of hGSTO1-1 with an IC_50_ of 4 µM [26]. Although soaking experiments with (+)-α-tocopherol succinate into crystals of hGSTO1-1 have not revealed the binding location (data not shown), it appears likely that it is congruent with the ligandin site described here. Binding of (+)-α-tocopherol succinate to hGSTO1-1 in crystals is most likely precluded by the limited solubility of the compound in crystallization solutions.

**Figure 6:**
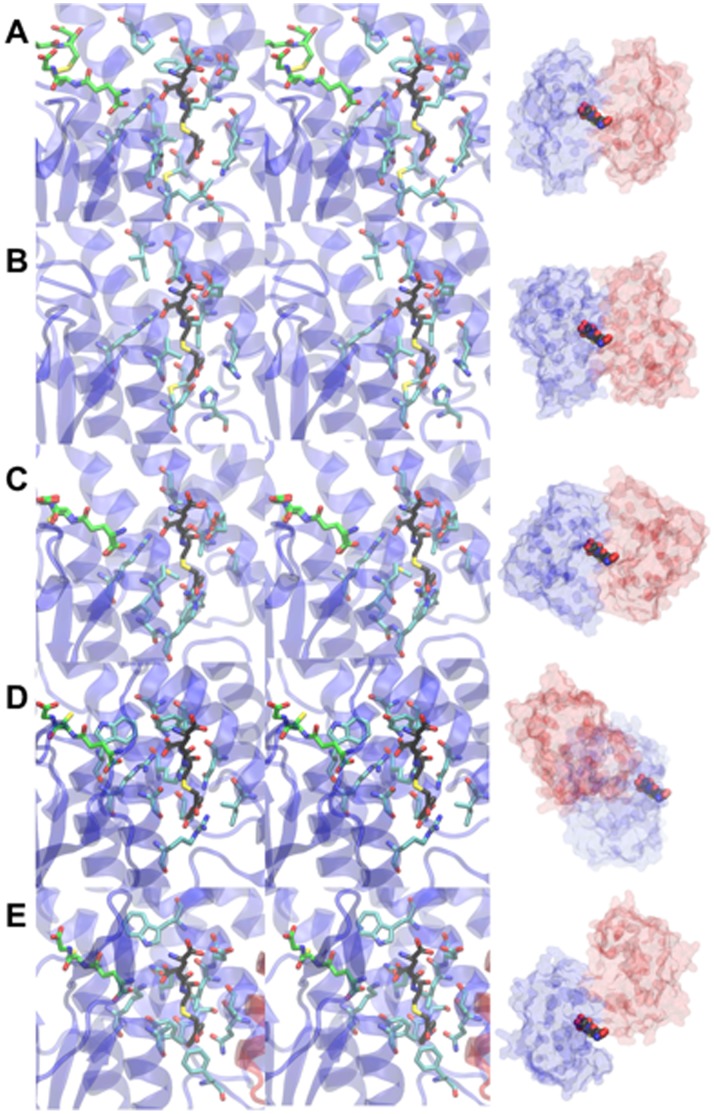
Comparison of the hGSTO1-1 L-site with related GSTs. Stereodiagrams of the L-site of hGSTO1-1 and equivalent sites in related GST are shown at left. Single monomers are shown in cartoon form with the second monomer omited for clarity. 4NPG (black carbon atoms), GSH (green carbon atoms) and L-site residues (or equivelent)(cyan carbon atoms) are shown in stick form. As a reference point, the model of 4NPG from the complex with hGSTO1-1 is shown overlaid in all structures. At right are shown transparent molecular surfaces of each GST with monomers in blue and red, and the model of 4NPG included as a reference point. The structures shown are (A) hGSTO1-1, (B) *Bombyx mori* GSTO3-3 (PDB 3RBT), (C) *Sphingobium* sp. SYK-6 LigG (PDB 4G10), (D) *Phanerochaete chrysosporium* GSTO3-3 (PDB ID 3PPU), (E) *Phanerochaete chrysosporium* GSTFuA (PDB 4G19).

It is instructive to compare the newly identified L-site with features in other omega-class and related GSTs. Residues in hGSTO1-1 binding the 4-Nitrophenacyl moiety are conserved or conservatively substituted in homologues from other species, and in hGSTO2 [10] but not more distantly related sequences (Figure 5). Recently described GSTs related to hGSTO1-1 may contain putative L-sites at identical locations. These include *Bombyx mori* GSTO3-3 [27], *Sphingobium* sp. SYK-6 LigG [28], *Phanerochaete chrysosporium* GSTO3-3 [29] and *Phanerochaete chrysosporium* GSTFuA [30]. While regions in the *Bombyx mori* GSTO3-3 and *Sphingobium* sp. SYK-6 LigG equivalent to the hGSTO1-1 L-site appear more occluded (Figure 6B, C), ligandin activity at these sites cannot be ruled out. The situation in *Phanerochaete chrysosporium* GSTO3-3 and GSTFuA is significantly altered due to the fundamentally different nature of dimerization interactions in these GSTs: the putative L-site regions are no longer on the dimer interfaces and are more solvent exposed (Figure 6D, E). It is noteworthy that ligandin activity has been reported in GSTFuA1. This GST binds 8-anilo-1-naphthalenesulfonicacid (8ANS) noncompetitively with substrates expected to bind in the H-site, but competitively with respect to GSH, and it has been proposed that the L-site in this GST co-localizes with the G-site [30].

Like the Omega-class GSTs, the beta-class GSTs from bacteria feature an active-site cysteine that forms a mixed disulfide with GSH, as demonstrated in the crystal structure of the *Proteus mirabilis* enzyme [31]. Therefore, in common with the Omega-class GSTs, binding of a second GSH molecule is a necessary physiological step for the regeneration of the reduced enzyme. Binding of GSH in the H-site of the *Ochrobactrum anthropi* beta-class GST has been observed [32], however, a mixed disulfide between the *Ochrobactrum anthropi* beta-class GST and GSH has not yet been observed [33], so the function of this second GSH-binding phenomenon remains an open question. This is not the case for Omega-class GSTs, where well-defined catalytic reactions result in oxidation of the enzyme, which must then be reduced. The H-sites of beta- and omega-class GSTs differ substantially and this is reflected in the distinct modes of binding of GSH. The second GSH molecule in the H-site of hGSTO1-1 is relatively disordered, with no specific hydrogen bonding interactions between enzyme and substrate. This implies that there is little specificity for GSH in this part of the reaction. Indeed, β-mercaptoethanol can substitute for the second GSH molecule in the regeneration of hGSTO1-1 [14]. The rearrangement of the H-site to accommodate the second GSH molecule helps explain the slower rate of reaction with this compound as reducing agent relative to β-mercaptoethanol, which is smaller and would appear less likely to require shifts in H-site residues in order to bind. From the structure, possible mechanisms for the activation of the second GSH molecule can be proposed. The backbone amide nitrogen group of F34 (in the active-site “CPFA loop”) is the only moiety that could potentially donate a hydrogen bond to the sulfur atom of the second GSH molecule. Although the NH to S distance in the complex with GSSG is 4.6 Å, this could plausibly be shorter prior to GSSG formation. Furthermore, the distribution of positive charges in the H-site (along with the dipole moment of helix α2 over which the sulfhydryl would be positioned) will favour deprotonation of the second GSH molecule, which can then attack the mixed disulfide. It is noteworthy that experimentally determined structures of glutaredoxins and thioltransferases contain loops structurally equivalent to the CPFA loop in hGSTO1-1. For example, in the crystal structure of human thiol-transferase is an active site CPFC motif [34] structurally analogous to CPFA in hGSTO1-1. As glutaredoxins form mixed disulfides with GSH, and can be reduced by a second GSH molecule with the formation of GSSG [35], this points to a conserved role for the active-site loop and possibly of the backbone F34 amide in activating thiol groups for enzyme reduction.

### Conclusion

A snapshot of hGSTO1-1 in the process of being regenerated has been observed by crystallography. We show that a GSSG molecule can bind in the active site, with one half of the molecule in the canonical G-site, and the other half in the H-site. There are few specific interactions of the glutathionyl moiety bound in the H-site. This apparent lack of specificity gives a possible explanation as to why other sulhydyl containing compounds can substitute for GSH in the recycling of oxidized hGSTO1-1. We have further identified a potential non-catalytic ligand-binding site in the dimer interface that may be the binding location of uncompetitive inhibitors such as tocopherol.
